# ^1^H NMR-Based Metabolomics Investigation of Copper-Laden Rat: A Model of Wilson’s Disease

**DOI:** 10.1371/journal.pone.0119654

**Published:** 2015-04-07

**Authors:** Jingjing Xu, Huaizhou Jiang, Jinquan Li, Kian-Kai Cheng, Jiyang Dong, Zhong Chen

**Affiliations:** 1 Department of Electronic Science, Fujian Provincial Key Laboratory of Plasma and Magnetic Resonance, Xiamen University, Xiamen, 361005, P. R. China; 2 Anhui University of Chinese Medicine, Hefei, 230031, P. R. China; 3 Department of Bioprocess Engineering & Innovation Centre in Agritechnology, Universiti Teknologi Malaysia, Johor Bahru, 81310, Malaysia; University of Florida, UNITED STATES

## Abstract

**Background and Purpose:**

Wilson’s disease (WD), also known as hepatoleticular degeneration (HLD), is a rare autosomal recessive genetic disorder of copper metabolism, which causes copper to accumulate in body tissues. In this study, rats fed with copper-laden diet are used to render the clinical manifestations of WD, and their copper toxicity-induced organ lesions are studied. To investigate metabolic behaviors of ‘decoppering’ process, penicillamine (PA) was used for treating copper-laden rats as this chelating agent could eliminate excess copper through the urine. To date, there has been limited metabolomics study on WD, while metabolic impacts of copper accumulation and PA administration have yet to be established.

**Materials and Methods:**

A combination of ^1^HNMR spectroscopy and multivariate statistical analysis was applied to examine the metabolic profiles of the urine and blood serum samples collected from the copper-laden rat model of WD with PA treatment.

**Results:**

Copper accumulation in the copper-laden rats is associated with increased lactate, creatinine, valine and leucine, as well as decreased levels of glucose and taurine in the blood serum. There were also significant changes in p-hydroxyphenylacetate (p-HPA), creatinine, alpha-ketoglutarate (α-KG), dimethylamine, N-acetylglutamate (NAG), N-acetylglycoprotein (NAC) in the urine of these rats. Notably, the changes in p-HPA, glucose, lactate, taurine, valine, leucine, and NAG were found reversed following PA treatment. Nevertheless, there were no changes for dimethylamine, α-KG, and NAC as a result of the treatment. Compared with the controls, the concentrations of hippurate, formate, alanine, and lactate were changed when PA was applied and this is probably due to its side effect. A tool named SMPDB (Small Molecule Pathway Database) is introduced to identify the metabolic pathway influenced by the copper-laden diet.

**Conclusion:**

The study has shown the potential application of NMR-based metabolomic analysis in providing further insights into the molecular mechanism underlying disorder due to WD.

## Introduction

Wilson’s disease (WD) also known as hepatoleticular degeneration (HLD) was first described by Wilson in 1912 [1]. It is an autosomal recessive illness characterized by excessive accumulation of copper in the liver, brain and other tissues [2,3]. The lifetime prevalence is estimated at 1:30,000, but a recent study of abnormal gene frequency has shown that the prevalence is getting higher (1:7026) [4]. WD is attributed to a defect in the ATP7B gene (on chromosome 13) which encodes the ATP-dependent copper transporting transmembrane protein mainly expressed in the liver. A defect in the ATP7B function leads to the accumulation of copper, primarily in the liver and subsequently in the brain and other organs. As a result of the deposits, there will be various clinical manifestations in the forms of hepatic and neuro-psychiatric features, such as cirrhosis, decreased serum ceruloplasmin and detectable Kayser-Fleischer rings, etc. [5,6].

The treatment for WD has advanced from an intramuscular administration of dimercaprol, a chelating agent (known as British anti-lewisite) to the more easily administered oral penicillamine (PA) which started in 1956 [7]. Subsequently, an alternative agent, trientine [8] was developed to remove copper, specifically for patients with adverse reactions to PA. Besides that, zinc sulphate was developed specifically to inhibit intestinal copper absorption and increase the capacity of metallothione of the binding copper in the liver, rather than the removal of the accumulated copper. [9]. Presently, PA is the first choice of drugs for WD treatment in China due to the preferred excretion effect of copper via urine although it is known to lead to numerous side effects [10]

Previous studies on WD have focused on aspects of molecular biology, protein, biochemistry and pathology[11]. However, different clinical manifestations of symptoms in WD patients and the pathological mechanism of organ lesions have yet to be explained. Comprehensive examinations of metabolic patterns of biological samples from WD patients with or without drug treatment are crucial for explaining the metabolic response to copper toxicity which can be done through a metabolomics analysis. NMR-based metabolomics combines NMR spectroscopy with pattern recognition techniques and quantitatively measures the multiparametric metabolic response to the changes in both endogenous and exogenous factors of integrated living systems. [12–15]. This analysis provides insights into the integrated function of the complex biosystem at the molecular level leading to a better understanding of the changing endogenous metabolic profile resulting from toxic insult [16], physiological challenge [17–19] or genetic modifications [20,21].

In this study, rats were fed with copper-laden diet to model the behavior of copper deposits in their organs. Using a high resolution ^1^H NMR spectroscopy, the biochemical variations of urine and serum from these experimental copper-laden rats were investigated. Then, PA was selected to treat the copper-laden rats based on preferred copper clearance and drug-induced variations in their biological metabotypes. This procedure has provided a novel insight into the systematic metabolism disrupted by abnormal copper deposits. Results of this study have validated the applicability of NMR-based metabolomics in the study of Wilson’s disease and may provide useful insights into the efficacy of using PA in copper-laden rats.

## Methods and Materials

### Animal handling and sample collection

Twenty-four healthy male Wistar rats (180±20g weight) were provided by Anhui Medical University. All rats were housed individually in metabolic cages. The animal room was maintained under controlled condition (a 12-hour light-dark cycle with constant temperature and humidity). The animals were provided with food and water ad libitum. Animal care and experimental procedures were approved and performed according to the guidelines of Animal Care and Use Committee of Anhui Medical University (Permit Number: AMU42-050609). All efforts were made to minimize animal suffering. After acclimatization for one week, these rats were randomly separated in three groups, i.e. control group (n = 8), copper-laden model group (n = 8), as well as copper-laden rats treated with PA (model+PA group, n = 8).

The control group was given normal diet and clean water throughout the experiment, while the model groups were fed with copper-laden diet (1g/kg CuSO_4_ and free access to 0.185%CuSO_4_ dissolved in the tap water) for 60 days [22].The model+PA group was administrated identically to the copper-laden rats except that the PA was given by 100 mg/kg body weight for latter the 30 days. After 60 days, the model group was proven to be a mature copper-laden model following examination of urinary and hepatic copper concentrations.

After 60-days of feeding, a 24-hour urine samples of all rats were collected from the metabolism cage. Whole blood was drawn from carotid arteries using a catheter and the blood was left to clot at room temperature for 1 h. Samples were centrifuged (3000 rpm for urine, 6000 rpm for serum) for 10 min at 4°C to remove particulate contaminants and then stored at -80°C until further analysis. The copper concentration in the urine and liver was examined by atomic absorption spectrophotometry (HITACHI Z-5000).

### Sample preparation and ^1^H NMR spectroscopy

For ^1^H-NMR experiment, 400 μL of blood or urine samples was mixed with 200 μL phosphate buffer solution (0.2M Na_2_HPO_4_/NaH_2_PO_4_, pH 7.4, 99.9% D_2_O) to reduce pH variation across samples. In addition, 0.3 mM TSP (3-(trimethylsilyl)propionic-2,2,3,3-d4 acid sodium salt) was used as an internal reference standard at δ 0.0. The mixture was then transferred into 5 mm NMR tube. The ^1^H NMR measurements of the urine and serum samples were performed using Varian NMR system 500 MHz spectrometer equipped with a triple resonance probe. The experimental temperature was set to 298 K and the 90° pulse length was calibrated individually for each sample.

For the urine samples, a conventional presaturation pulse sequence for solvent suppression based on the 1D version of NOESY pulse sequence known as NOESYPR (Nuclear Overhauser Effect SpectroscopY with PResaturation, delay-90°-t1-90°-tm-90°-acquisition) was used [23], where the t_1_ represented the first increment in the NOESY experiment and was set to 2 μs; a weak irradiation on water signal was applied to suppress solvent during the mixing time of 120 ms and recycle delay of 2 s. For the blood serum samples, an additional Carr-Purcell-Meiboom-Gill (CPMG) spin-echo pulse train [24] was incorporated into the NOESYPR sequence with a relaxation time (2nτ) of 100 ms and an echo time (τ) of 250 μs. A total of 256 scans with a spectral width of 10 kHz were collected for all NMR experiments. The acquired signals were zero filled to 32k before Fourier transformation (FT). For data analysis, metabolites in the urine and serum ^1^H NMR spectra were assigned with reference to published data [25] and HMDB database (http://www.hmdb.ca/).

### Data preprocessing of NMR spectra and pattern recognition

The collected NMR spectra were phased and baseline corrected using the software MestRe-C 4.8 (http://www.mestrec.com). Then baseline correction was refined using the autocorrection algorithm, proposed by Xi and Rocke [26]. All NMR spectra were also peak-aligned manually using in-house software to minimize variation due to peak shift [27]. The chemical shift regions of δ 4.6–6.0 (water and urea resonances), δ 0.0–0.2 (TSP resonance) and the regions which are peak-free for all spectra were excluded from further analysis. S1 Table and S3 Table show the raw spectral data of serum and urine samples respectively. Then each spectrum was binned into segments by adaptive binning method [28]. To account for variations in sample concentration, the spectra were normalized using group aggregating normalization technique [29]. S2 Table and S4 Table show the normalized bucket data of serum and urine samples respectively.

The NMR spectral data were converted to Microsoft Excel format and imported into SIMCA-P software (version 12.0, Umetrics AB, Umeå, Sweden) for multivariate analysis. Partial least squares discriminant analysis (PLS-DA) is a regression method that uses binary variables Y to indicate class membership. [30]. The PLS-DA projects the original variables X (metabolic concentrations) to latent variables that focus on class separation, thus obtaining a better classification. The quality of the models is described by R^2^X and Q^2^ values. R^2^X is defined as the proportion of variance in the data explained by the models and indicates goodness of fit, and Q^2^ is defined as the proportion of variance in the data predictable by the model and indicates predictability [31].

The metabolites with high Variable importance in the projection (VIP) values in each PLS-DA models are selected to further investigate their quantitative changes in all three studied groups. The selected metabolites were then assessed using unpaired t-test (OriginPro ver. 8.1).

## Results and Discussion

### Determination of copper accumulation

As shown in Table 1, the copper contents in 24h urine and unit mass of liver are higher in the copper-laden model group, as compared to the control group. This shows that a 60-day copper-laden diet had produced an effective animal model for copper accumulation. After PA injections were administered for 30 days, the copper deposits in the liver were found to have significantly decreased, but copper excretion had increased in the urine. The current result is consistent with previous findings showing that PA has promoted urinary excretion of copper. The detailed metabolic changes induced by copper-laden diet and PA treatment were then investigated using NMR-based metabolomics.

**Table 1:** Copper contents of control group, copper-laden model group and model+PAgroup.

Copper content	Control	Copper-laden Model	Model+PA
Urinary copper (μg/24h)	36.98±6.72	156.83±34.03*	380.31±46.08*
Hepatic copper (μg/g)	5.54±0.74	108.15±12.60*	43.84±19.41*

Note

* *p*<0.05

### PLS-DA of urine and blood serum samples

**Fig 1:**
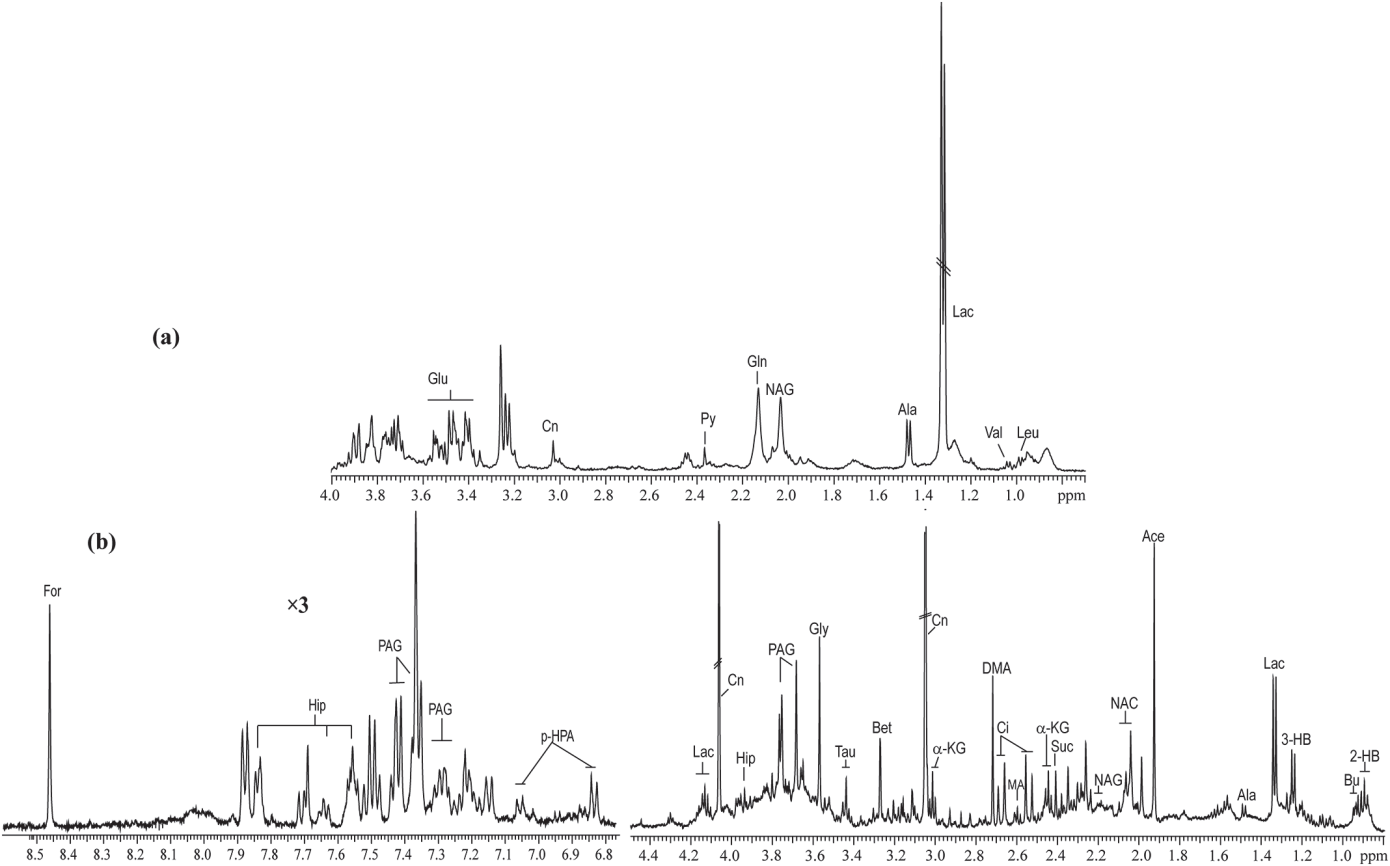
Typical ^1^H NMR spectra of serum (a) and urine (b) samples obtained from the WD rat model. Leu: leucine; Val: valine; Lac: lactate; Ala: alanine; NAG: N-acetylglutamate; Gln: Glutamine; Py: pyruvate; Cn: creatinine; Glu: glucose; 2-HB: 2-hydroxybutyrate; Bu: butyrate; 3-HB: 3-hydroxybutyrate; Ace: acetate; NAC: N-acetylglycoprotein; Suc: succinate; a-KG: alpha-ketoglutarate; Ci: cirtrate; MA: methylamine; DMA: dimethylamine; Bet: betaine; Tau: taurine; Gly: glycine; PAG: phenylacetylglycine; Hip: hippurate; p-HPA: para-hydroxyphenylacetate; For: formate.

**Fig 2:**
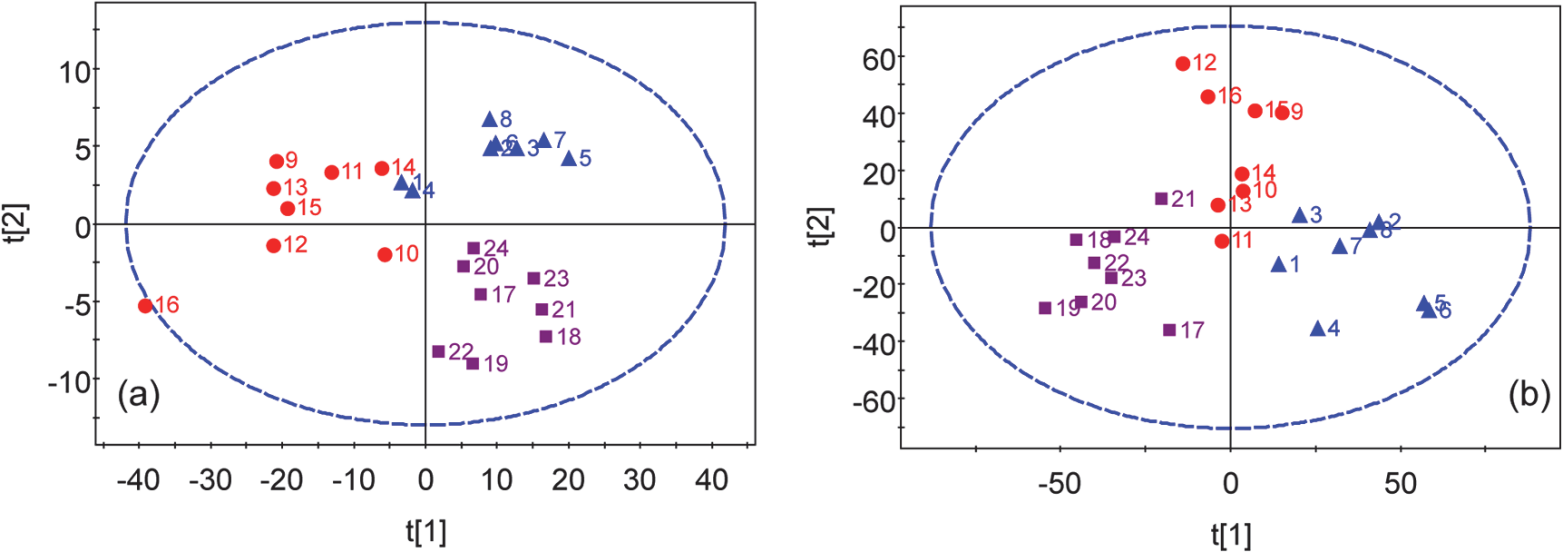
PLS-DA models of ^1^H NMR metabolomics data for control group (Blue triangle), copper-laden model group (Red dot) and model+PA group (Purple square). (a) scores plot of ^1^H-NMR data of blood serum samples. (b) scores plot of ^1^H-NMR data of urine samples.

Typical ^1^H NMR spectra of serum and urine taken from copper-laden rats are shown in Fig. 1. PLS-DA analysis of either urine or blood serum samples showed that the samples clustered based on studied groups, which are the controls, copper-laden model and model+PA groups (Fig. 2). To investigate further the metabolic patterns and identify potential characteristic metabolites induced by the copper-laden diet and PA treatment, two-group comparisons were carried out using PLS-DA.

### Analysis of blood serum metabolic profiles

**Fig 3:**
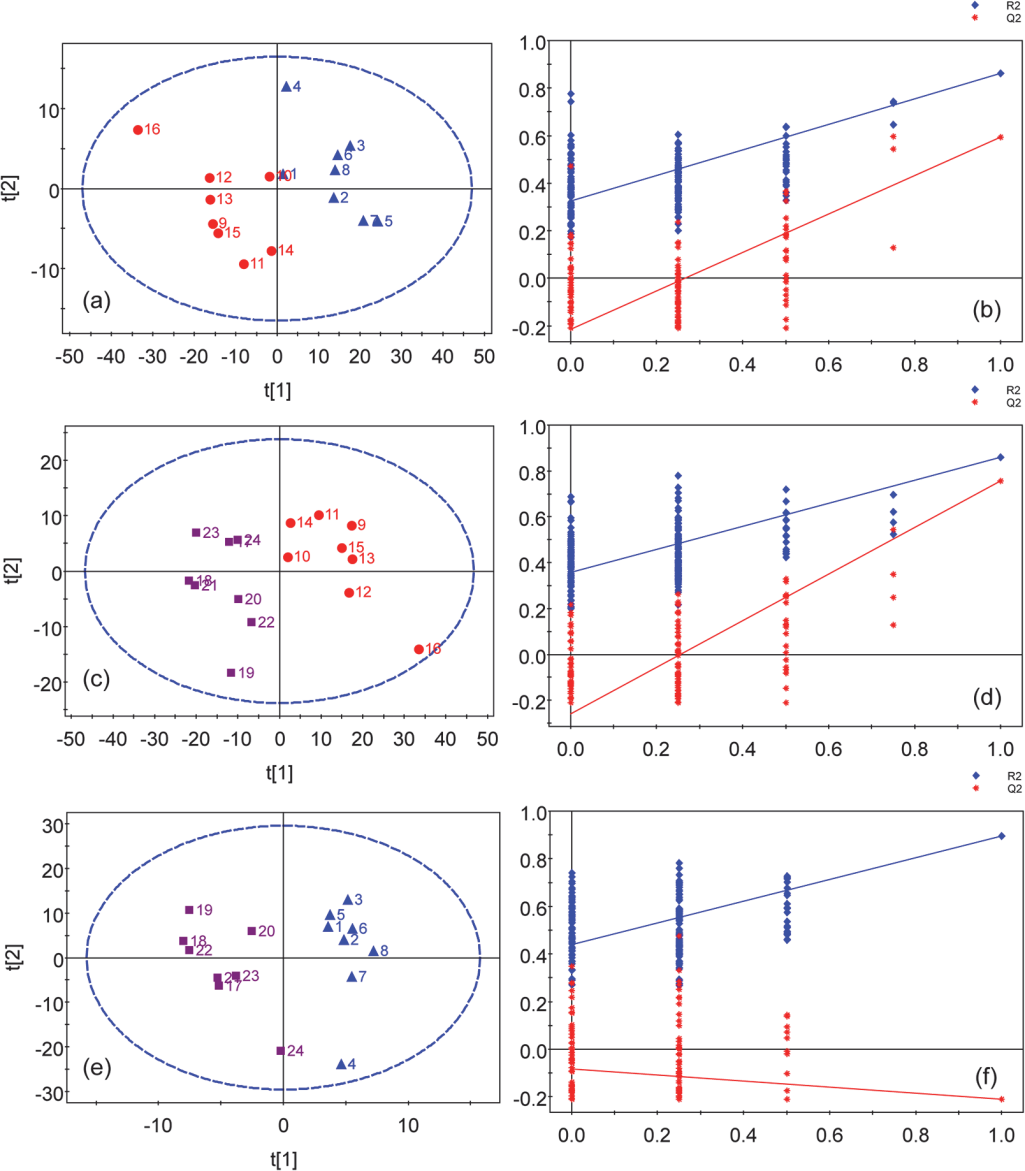
PLS-DA models of ^1^H NMR serum spectra. (a) scores plot of the control and model groups(R^2^ = 71.2%, Q^2^ = 62.9%) with one PLS-DA component and (b) permutation test for model in (a); (c) scores plot of the copper-laden model and model+PA groups (R^2^ = 85.9%, Q^2^ = 75.7%) with 2 components and (d) permutation test for model in (c); (e) scores plot of the control and model+PA groups (R^2^ = 90.4%, Q^2^ = 21.0%) with one component and (f) permutation test for model in (e). Colored symbols: Blue triangle for the control group; Red dot for the model group; Purple square for the model+PA group. Blue diamond is denoted as R^2^; Red star is denoted as Q^2^. The ellipse shows the Hotelling’s T2-range (at a significance level of 0.05).

The PLS-DA of blood serum data showed the group separation between the controls and copper-laden rats (Fig. 3A) and between copper-laden model rats and model+PA group (Fig. 3C). The corresponding validation plots of the two PLS-DA models in Fig. 3B and 3D suggest that both models appear to be robust. Although the controls and model+PA groups separated from each other in the scores plot as in Fig. 3E, this PLS-DA model has failed in the model validation. Therefore, the group classification of this model is unreliable and was not used for further analysis. The VIP values obtained from a PLS-DA model are used as parameters for variable selection. A variable with a VIP value greater than one is considered important and picked out to be investigated for its statistical significance between groups by unpaired t-test.

**Fig 4:**
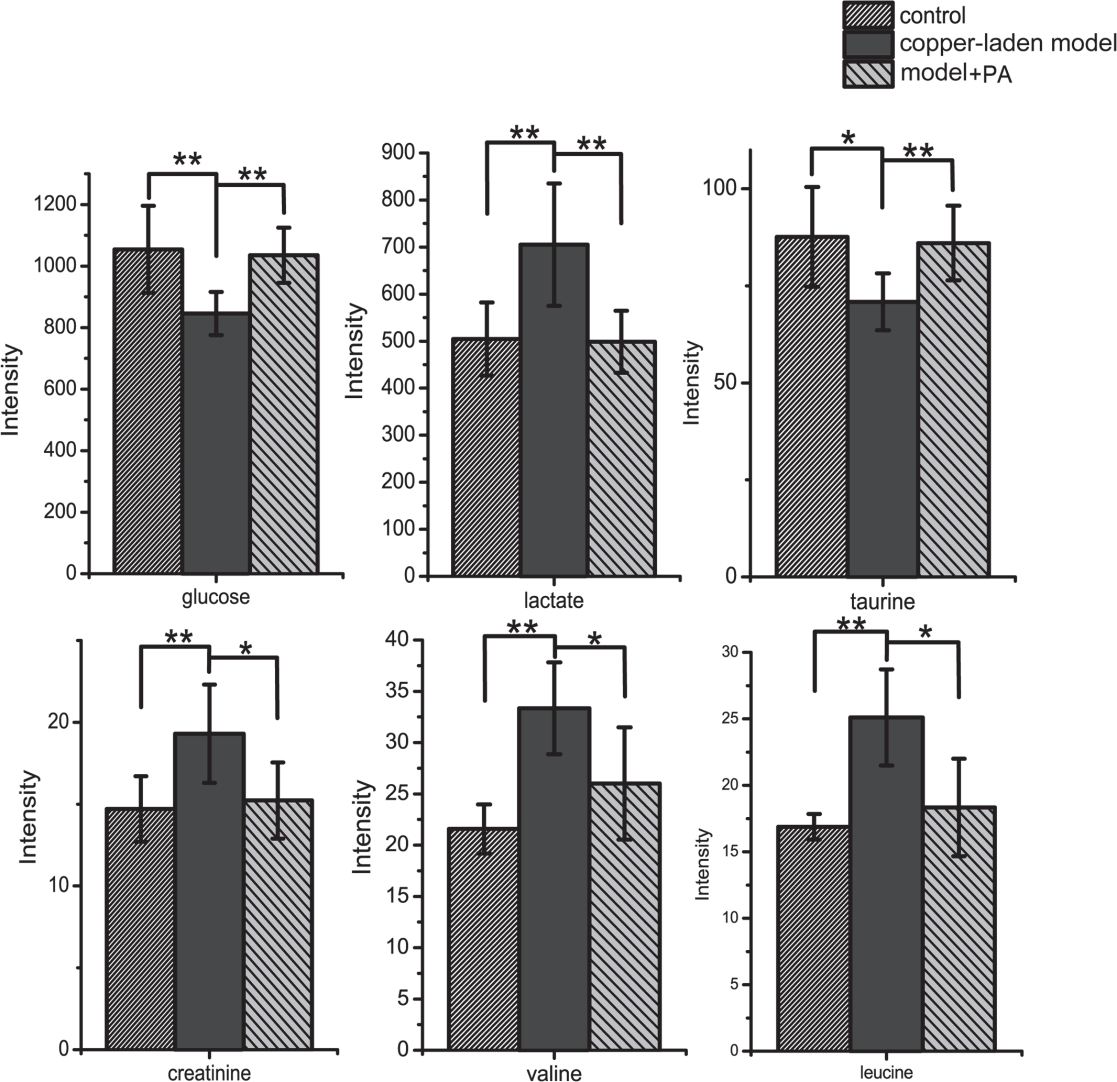
Comparison of serum metabolites between control, copper-laden model and model+PA groups. Results are presented as mean ± S.D. (n = 8). * *p*< 0.05; ** *p*<0.01.

The blood glucose level, tightly regulated in body is an important part of metabolic homeostasis. In the present study, the level of serum glucose is decreased due to the copper-laden diet, but the glucose concentration can be restored by PA treatment (Fig. 4). The reason for this phenomenon is due to the following conditions. Firstly, glucose is absorbed from the intestines via bloodstream, and the hormone insulin helps to make it available for cell utilization. Previous research has reported that the gastrointestinal distress would block intestinal absorption process [32]. Secondly, copper is incorporated into a number of metalloenzymes including mitochondrial oxidative phosphorylation such as cytochrome c oxidase which is a terminal enzyme of the mitochondrial respiratory chain. Although the detailed mechanism is still unclear, the copper deposition could have promoted glycoxidation process as well as lipid peroxidation [33]. Thus, the level of serum glucose is decreased. Thirdly, the glycogen synthase is the next important step in regulating glycogen metabolism and glucose storage. The enzyme activity of glycogen synthase are known to be phosphorylated and inactivated by glycogen synthase kinase 3 (GSK-3), protein kinase A (PKA) and AMP-activated protein kinase (AMPK) [34]. The increased copper deposits has resulted in dose-dependent inhibition of GSK and AMPK, and an indirect activation of glycogen synthase, which promotes glycogen synthesis leading to a reduction in blood glucose[35]. While copper-laden rats were treated with PA, this ‘decoppering’ drug had promoted urinary excretion of copper and normalized the free copper concentration in the blood to reduce copper poisoning. It is hypothesized that such a treatment may regulate glycoxidation process induced by the laden copper diet by raising blood glucose production.

The most notable metabolic changes in the blood serum of the copper-laden rats include the upregulation of lactate and branched chain amino acids such as valine and leucine. The accumulated copper has enhanced the glucose consumption and lactate production by stimulating glycolysis and inhibiting the aerobic respiration [36,37]. The presence of elevated lactate is present in patients with mitochondrial disorders such as Kearns-Sayre syndrome, mitochondrial myopathy, encephalopathy and lactic acidosis. In WD patients, the gene defect (P-ATPase) affects the function of cytochrome C oxidase at the level of the mitochondria thus inhibits the aerobic metabolism in mitochondria but enhances anaerobic metabolism resulting in the elevation of lactate.

With the development of WD, deficiency of the enzyme of alpha-keto acid dehydrogenase complex will inhibit the metabolism of these amino acids [38]. Upregulation of the branched chain amino acids (BCAAs: valine, leucine) indicate that the accumulated copper has inhibited the enzyme activity of 3-hydroxyisobutyryl-CoA hydrolase and damaged the valine catabolism [39]. In addition, the degradation of cytotoxic methacrylyl-CoA which reacts highly with thiol group is important to the liver [40]. The disruption of methacrylyl-CoA disposal is probably due to liver failure in this animal model of Wilson’s Disease [41].

Taurine is an abundant intracellular organic acid and a major constituent of bile. It is synthesized mainly in the cysteine sulfinic acid pathway in the pancreas. The sulfhydryl group of cysteine is oxidized to cysteine sulfinic acid which turns is decarboxylated to hypotaurine, later oxidized to become taurine. Taurine or glycine forms conjugate with bile acids via its amino terminal group with chenodeoxycholic acid and cholic acid to form bile salts, enhancing bile flow and clearance of cholesterol by the liver. Taurine is involved in many physiological processes, namely osmoregulation [42], clearance of toxic intermediates [43], and calcium mobilization[44]. However, the exact role of taurine in these processes is still not clear. In this study, the decreased level of taurine in serum was deemed to be the outcome of over-secretion of bile into the duodenum. Additionally, taurine has been shown to reduce the secretion of apolipoprotein B100 and lipids, which are components of VLDL and LDL [45]. Studies also shown that taurine decreased the blood glucose levels in animals [46]. However, clinical studies have also illustrated that taurine had no effect on insulin secretion or sensitivity [47]. Thus, the action of taurine on glucose and lipid metabolism requires further investigation.

### Analysis of urinary metabolic profiles

**Fig 5:**
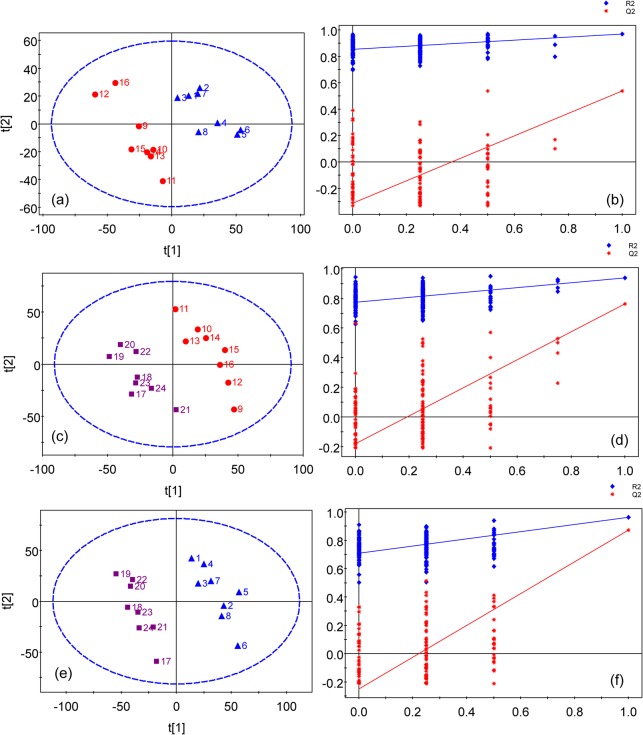
PLS-DA scores plots of ^1^H NMR urine spectra. (a) Scores plot of the control and copper-laden model groups (R^2^ = 92.6%, Q^2^ = 57.9%) with 2 components and (b) permutation test for model in (a); (c) scores plot of the copper-laden model and model+PA groups (R^2^ = 98.1%, Q^2^ = 82.6%) with 3 components and (d) permutation test for model in (c); (e) scores plot of the control and model+PA groups (R^2^ = 98.9%, Q^2^ = 87.9%) with 2 components and (f) permutation test for model in (e); Colored symbols: Blue triangle for the control group; Red dot for the model group; Purple square for the model+PA group. Blue diamond is denoted as R^2^; Red star is denoted as Q^2^. The ellipse shows the Hotelling’s T2-range (at a significance level of 0.05)

**Fig 6:**
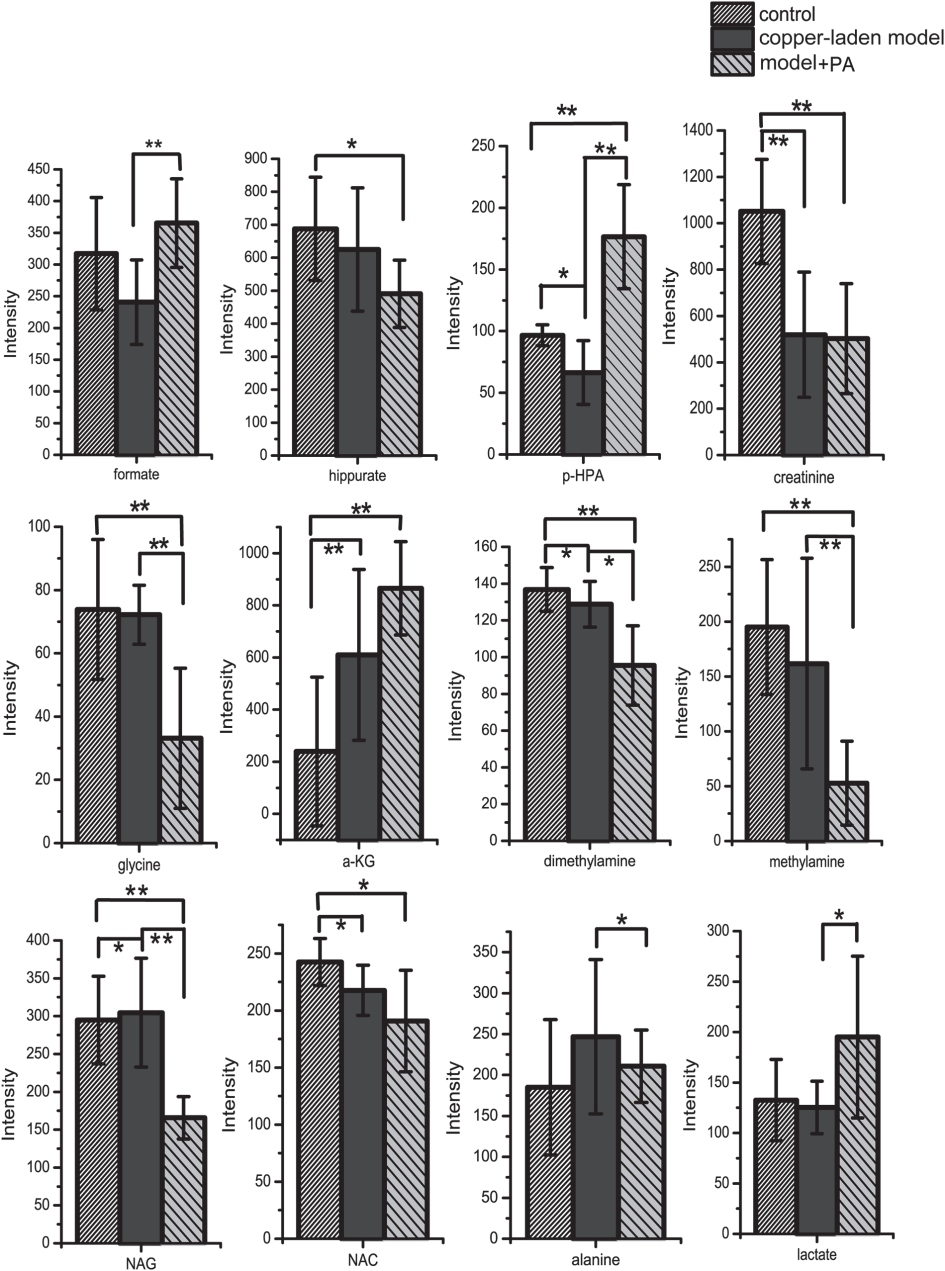
Comparison of urinary metabolites between control, copper-laden model and model+PA groups. Results are presented as mean ± S.D. (n = 8). * *p*< 0.05; ** *p*<0.01. p-HPA: p-hydroxyphenylacetate, α-KG: alpha-ketoglutarate, NAG: N-acetylglutamate, NAC: N-acetylglycoprotein.

The PLS-DA scores plots of the urine samples showed that there are good separations between the copper-laden and the control group (Fig. 5A), the model+PA and the copper-laden model group (Fig. 5C), and model+PA and the control group (Fig. 5E). Satisfactory permutation test plots were obtained for all three PLS-DA models (Fig. 5B, 5D and 5E). Then, metabolites that contributed significantly to group classification were identified from these PLS-DA models (Fig. 6). In addition, the P values indicated in the plots are meant to highlight the significant differential metabolites between the different groups.

**Fig 7:**
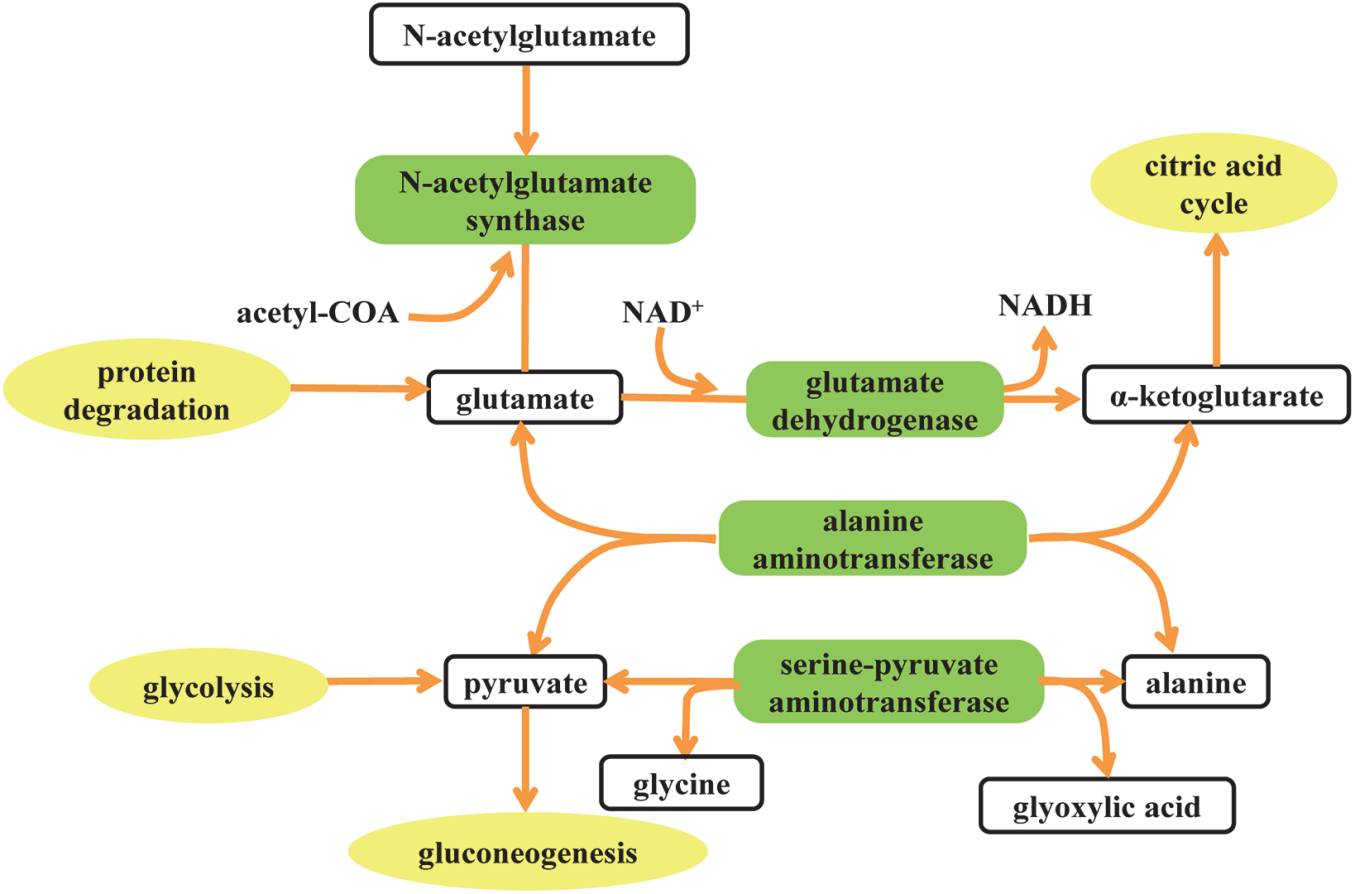
Metabolic pathways affected by copper accumulation. Elements in blank box represent the metabolites influenced by copper-laden diet and PA treatment. The elements in green box represent the enzymes involved in the metabolic pathway. The elements in yellow box represent the peripheral pathways that can be associated with the characteristic metabolites.

The metabolic changes induced by copper laden and PA treatment in model rats were then characterized. It is obvious that p-hydroxyphenylacetate (p-HPA), creatinine, alpha-ketoglutarate (α-KG), dimethylamine, N-acetylglutamate (NAG), N-acetylglycoprotein (NAC) are found to have changed in concentration after being fed with copper-laden diet. In addition, the amount of formate, p-HPA, glycine, dimethylamine, methylamine, NAG, alanine and lactate are found to be perturbed by PA treatment. The results of the treatment when compared with that of the control group showed that PA could have affected the basal metabolism as concentrations of hippurate, p-HPA, creatinine, glycine, dimethylamine, methylamine, NAG and NAC have changed. The reasons for such alterations are found by inferring from literature. Furthermore, a tool named SMPDB (Small Molecule Pathway Database, www.smpdb.ca) designed specifically to support pathway elucidation and discovery in metabolomics, transcriptomics, proteomics and systems biology has been used to intuitively interpret the perturbed metabolic pathway (Fig. 7).

After being fed with copper, urinary excretions of α-KG and NAG were found to have increased. Alpha-KG, or known as oxo-glutarate is a key intermediate in TCA cycle. It has two main functions; one is to combine with ammonia to form glutamate and then glutamine, and the other is to combine with nitrogen released in the cell to prevent nitrogen overload. The metabolite α-KG can be produced by oxidative deamination of glutamate via glutamate dehydrogenase. Furthermore, glutamate and acetyl-CoA can be biosynthesized into NAG by the enzyme N-acetylglutamate synthase. The presence of copper may facilitate the formation of reactive oxygen species, and inhibited pyruvate dehydrogenase and α-KG dehydrogenase in vitro and in animal models of Wilson’s disease in vivo [48]. Alpha-ketoglutarate dehydrogenase (KGDHC) complex activity is diminished in a number of neurodegenerative disorders such as in Alzheimer Disease (AD) because the reduced activity is believed to contribute to the major loss of cerebral energy metabolism that accompanies this disease [49]. The reaction of decarboxylation of α-KG which is inhibited in copper-laden group has caused α-ketoglutarate to be deposited and excreted in the urine. Thus, the increased N-acetylglutamate may be the result of the excess production prompted by the elevated level of α-ketoglutarate in the α-ketoglutarate-glutamate-N-acetylglutamate pathway.

Alanine plays a key role in glucose-alanine cycle between tissues and the liver. In muscle and other tissues that degrade amino acids for fuel, amino groups are collected in the form of glutamate by transamination. Glutamate can then transfer its amino group through the action of alanine aminotransferase to pyruvate, a product of muscle glycolysis, forming alanine and α-KG. The activity of alanine aminotransferase in blood serum was shown to be significantly elevated with the onset of fulminant hepatitis in rats subjected to WD [50]. Notably, mRNA of *Srebf1* was down regulated during fulminant hepatitis. In the study, copper accumulation in the liver could have modulated the liver function and led to hepatic inflammation in the early phases of liver damage. The transamination reaction involving the interconversion of alanine and pyruvate, coupled with the interconversion of glyoxylate and glycine may have been disrupted, although it should be noted that any variation in concentration of alanine and glycine is beyond the detection sensitivity of metabolomics technique based on NMR spectra.

## Conclusion

In the current study, metabolomics has been used to highlight the metabolic impact of copper-laden diet and effect of PA treatment in the copper-laden rat model. NMR-based metabolomics has been shown to be an efficient technique to investigate the metabolic perturbation due to copper accumulation and PA treatment. The changes of endogenous metabolites in urine and serum samples from models of copper-laden rats, model+PA and control rats were identified by PLS-DA. The toxicity of accumulated copper is proven to be selective and has affected a number of metabolic pathways. A comparison between the current findings and zinc therapy warrants future investigation, as treatment with zinc was found to normalize the serum free copper concentration [51]. To complement the current study, future proteomic and immunohistochemistry studies may provide further insights into the metabolic mechanism and discover more effective treatments for WD. In summary, the current results indicate that the ^1^H NMR-based metabolomics has the potential to uncover the metabolic response of copper toxicity that could provide better a understanding of the metabolic effect and efficacy of drug treatments.

## Supporting Information

S1 Table Raw Spectral Data of Serum Samples. (XLSX)

S2 Table Normalized Bucket Data of Serum Samples. (XLSX)

S3 Table Raw Spectral Data of Urine Samples. (XLSX)

S4 Table Normalized Bucket Data of Serum Samples. (XLSX)
